# The multifaceted roles of metabolic enzymes in the *Paracoccidioides* species complex

**DOI:** 10.3389/fmicb.2014.00719

**Published:** 2014-12-19

**Authors:** Caroline M. Marcos, Haroldo C. de Oliveira, Julhiany de F. da Silva, Patrícia A. Assato, Ana M. Fusco-Almeida, Maria J. S. Mendes-Giannini

**Affiliations:** Laboratório de Micologia Clínica, Departamento de Análises Clínicas, Faculdade de Ciências Farmacêuticas, Universidade Estadual PaulistaAraraquara, Brazil

**Keywords:** *Paracoccidioides* spp., moonlighting proteins, virulence, glycolytic pathway and tricarboxylic acid cycle, glyoxylate cycle, adhesins

## Abstract

*Paracoccidioides* species are dimorphic fungi and are the etiologic agents of paracoccidioidomycosis, which is a serious disease that involves multiple organs. The many tissues colonized by this fungus suggest a variety of surface molecules involved in adhesion. A surprising finding is that most enzymes in the glycolytic pathway, tricarboxylic acid (TCA) cycle and glyoxylate cycle in *Paracoccidioides* spp. have adhesive properties that aid in interacting with the host extracellular matrix and thus act as ‘moonlighting’ proteins. Moonlighting proteins have multiple functions, which adds a dimension to cellular complexity and benefit cells in several ways. This phenomenon occurs in both eukaryotes and prokaryotes. For example, moonlighting proteins from the glycolytic pathway or TCA cycle can play a role in bacterial pathogenesis by either acting as proteins secreted in a conventional pathway and/or as cell surface components that facilitate adhesion or adherence. This review outlines the multifunctionality exhibited by many *Paracoccidioides* spp. enzymes, including aconitase, aldolase, glyceraldehyde-3-phosphate dehydrogenase, isocitrate lyase, malate synthase, triose phosphate isomerase, fumarase, and enolase. We discuss the roles that moonlighting activities play in the virulence characteristics of this fungus and several other human pathogens during their interactions with the host.

## INTRODUCTION

A great challenge in studying proteins is understanding how encoded proteins function and interact with each other to coordinate essential cellular processes. Although many protein roles can be inferred by homology-based function predictions, this approach may be complicated for multifunctional proteins. The notion that one gene encodes one protein and results in only one function is outdated because proteins may have multiple functions (including on a single polypeptide chain), and the function may change based on external signals (Kirschner and Bisswanger, 1976; Jeffery, 1999; Wolff and Arnau, 2002; Jeffery, 2009; Pandini et al., 2012; Wienkers and Rock, 2014).

Multiple binding sites or changes in unusable regions of a protein structure may produce a new function because many proteins seem larger than necessary to perform only one function at a single binding site. These multifunctional proteins may benefit an organism because synthesizing fewer proteins may save cellular energy for additional functions, such as growth and reproduction (Jeffery, 1999).

Moonlighting proteins are exceptional multifunctional proteins; these multifunctional proteins can perform several additional functions that are often unrelated. These functions are typically independent, which means that if one function is inactivated, due a mutation, for example, the second function is unaffected (Huberts and van der Klei, 2010). The function of a moonlighting protein can vary based on changes in cellular location or expression, cell type, association between two or more polypeptide chains and the cellular levels of a ligand, substrate, cofactor, product, or different binding sites (Jeffery, 2003a); moonlighting cannot be attributed to hybrid genes, which are single genes that code for multiple proteins or polypeptides that express different functions after protease cleavage (Kainulainen and Korhonen, 2014). Moonlighting functions have been demonstrated by multiple independent studies with unexpected phenotypes, locations, and binding partners (Copley, 2012).

The steady increase in new proteins characterized as multifunctional supports the potential importance of in-depth studies on the mechanism underlying these moonlighting functions in the same cell. (Chung et al., 1999; Jeffery, 1999, 2003a). Moonlighting may be due to joint engineering of communication and cooperation for various functions and paths in a complex cell or different cell types in an organism (Jeffery, 2003b).

Multifunctional proteins are present in prokaryotes and eukaryotes, such as mammals, which compound the protein arsenal of these organisms (Clarke et al., 2001; James and Viola, 2002; Brilli and Fani, 2004; Orita et al., 2005). The moonlighting activities of one protein are typically in addition to their role in chemical metabolic reactions, which demonstrates that these proteins are highly variable; metabolic enzymes can perform double duty as transcription factors, participate in assembly or autophagy, or maintain the levels of oxidative phosphorylation in the cells through maintaining mitochondrial DNA, among other functions (Chen et al., 2005; Gancedo and Flores, 2008). Intriguingly, in many cases, these proteins are constitutively expressed at low levels and act as enzymes, but when they are expressed at high levels, they perform moonlighting functions (Baker, 1991; Gómez et al., 2011).

Although highly conserved proteins perform many moonlighting functions, moonlighting functions cannot be predicted based on sequence and structural comparisons. Researchers speculate that evolution produced proteins with almost identical structures but different functions because moonlighting may provide a means to expand the functional capabilities of an organism without a genome-wide expansion (Kelkar and Ochman, 2013). Researchers have proposed that a protein must have some inherent compatibility for a new function to develop a moonlighting function (Aharoni et al., 2005).

To identify the moonlighting site or sites, we must first study how the moonlighting protein evolved and how the moonlighting function is related to the original “active site” (Henderson and Martin, 2011). Certain moonlighting proteins are recruited to the cell surface and involved with pathogenic processes (Pancholi and Chhatwal, 2003; Wang et al., 2013). This process not only occurs in bacteria but also in fungi, including the *Paracoccidioides* genus. Moonlighting proteins may relocate to the bacterial surface and present adhesion activities specific for host cell targets. These adhesive activities in moonlighting proteins have been widely studied and seem to play important roles in bacterial adhesion and colonization (Wang et al., 2013). Most abundant moonlighting adhesins are proteins that interact with the adhesion complex through binding fibronectin, which is a protein present at high concentrations in the fluids between cells and in the extracellular matrix (ECM), and it links cells to the ECM through specific transmembrane receptors (Henderson and Martin, 2011).

Examples of secondary functions associated with catalysis have been reported in many organisms, including plants (Moore, 2004), animals (Sriram et al., 2005), yeast (Gancedo and Flores, 2008), and prokaryotes (Jeffery, 1999, 2004). In bacteria, the glycolytic enzymes glyceraldehyde-3-phosphate dehydrogenase (GAPDH) and enolase (ENO) as well as the chaperonin 60, Hsp70, and peptidyl-prolyl isomerase most commonly exhibit moonlighting functions during bacterial virulence, such as adhesion and modulation of host cell signaling processes (Henderson and Martin, 2011). Most examples have been demonstrated in yeast (Gancedo and Flores, 2008) likely because it is the best understood and an extensively studied model organism. The known moonlighting functions are extremely diverse and involved in several biological functions. Examples of well-characterized moonlighting proteins in *Paracoccidioides* spp. and other fungal species are shown in **Table 1**.

**Table 1:** Moonlighting proteins characterized in different fungal species.

Proteins	Metabolic pathway or function	Moonlighting function	Fungal species	Reference
Enolase	Glycolysis	Thermal tolerance and growth control	*Saccharomyces cerevisiae*	Sekita et al. (1985)
		Invasion process; cell wall construction	*Candida albicans*	Walsh et al. (1991), van Deventer et al. (1994), Angiolella et al. (1996)
		Invasion process (plasminogen binding)	*C. albicans; Aspergillus fumigatus; Paracoccidioides* spp.	Eroles et al. (1997), Fox and Smulian (2001), Jong et al. (2003), Nogueira et al. (2010), Marcos et al. (2012)
		Adhesion process (fibronectin binding)	*Paracoccidioides* spp.	Donofrio et al. (2009), Marcos et al. (2012)
Malate synthase	Glyoxylate cycle	Adhesion process (type I and IV collagen and fibronectin binding)	*Paracoccidioides* spp.	da Silva Neto et al. (2009)
Aconitase	TCA cycle	Mitochondrial DNA maintenance	*S. cerevisiae*	Albring et al. (1977), Rouault et al. (1991), Lu et al. (2001), Chen et al. (2005), Myers (2009)
		Iron regulatory protein	*Paracoccidioides* spp., *S. cerevisiae*	Narahari et al. (2000), da Fonseca et al. (2001), Barbosa et al. (2004)
Aldolase	Glycolysis	Invasion process (plasminogen binding)	*C. albicans, Paracoccidioides* spp.	Burucoa et al. (1995), McCarthy et al. (2002), Pitarch et al. (2002), Crowe et al. (2003), Chaves (2013)
GAPDH	Glycolysis	Adhesion and invasion processes (fibronectin, laminin and plasminogen-binding)	*C. albicans*	Pancholi and Fischetti (1992), Gil-Navarro et al. (1997), Delgado et al. (2003), Starnes et al. (2009)
		Adhesion process (laminin, fibronectin and type I collagen binding)	*Paracoccidioides spp.*	Barbosa et al. (2006)
Isocitrate lyase	Glyoxylate cycle	Growth	*Aspergillus fumigatus*	Gainey et al. (1992), Valenciano et al. (1996), Ebel et al. (2006)
		Adhesion process (fibronectin and type IV collagen binding)	*Paracoccidioides* spp.	Cruz et al. (2011)
Triose phosphate isomerase	Glycolysis	Adhesion process (laminin and fibronectin binding)	*Paracoccidioides* spp.	da Fonseca et al. (2001), Pereira et al. (2004, 2007)

Many currently known moonlighting proteins are highly conserved enzymes present in many different organisms (Fothergill-Gilmore and Michels, 1993; Jeffery, 1999). Among these proteins, we highlight the enzymes involved in sugar metabolism (Hendriks et al., 1988; Wistow et al., 1988; Yuan et al., 1997; Chen et al., 2005; Decker and Wickner, 2006; Lu et al., 2007). Researchers have also suggested that most enzymes in the glycolytic pathway and tricarboxylic acid (TCA) cycle have moonlighting functions (Kim and Dang, 2005; Sriram et al., 2005). Moreover, Commichau et al. (2009) showed interactions between glycolytic enzymes and proteins involved in RNA degradation, which suggests the presence of additional moonlighting functions for these proteins.

In this review, we discuss multiple attributes of moonlighting proteins in *Paracoccidioides* spp. and indicate the studies that justify their inclusion in this category. Another goal of this review is to highlight the importance of this phenomenon and its wide implications for both basic and applied research.

## MOONLIGHTING *Paracoccidioides* SPECIES COMPLEX PROTEINS

Studies have characterized the ECM components involved in interactions between *Paracoccidioides* spp. and the host. This genus is composed of two species, *Paracoccidioides lutzii* and *P. brasiliensis*; the latter is sub-classified into three different phylogenetic groups. The large number of tissues that this fungus can colonize and infect suggests that it includes many cell adhesins. Certain molecules from *Paracoccidioides* spp. were identified as ligands for ECM components (Mendes-Giannini et al., 2008). Gp43 was the first molecule identified as a laminin-binding protein (Vicentini et al., 1994; Hanna et al., 2000). Additional binding affinity assays have shown that gp43 can bind both fibronectin and laminin. In *Paracoccidioides* spp, certain additional adhesins have also been described and may play an important role in pathogenesis (Andreotti et al., 2005; González et al., 2005; Barbosa et al., 2006; Mendes-Giannini et al., 2006; Pereira et al., 2007; Donofrio et al., 2009).

**FIGURE 1:**
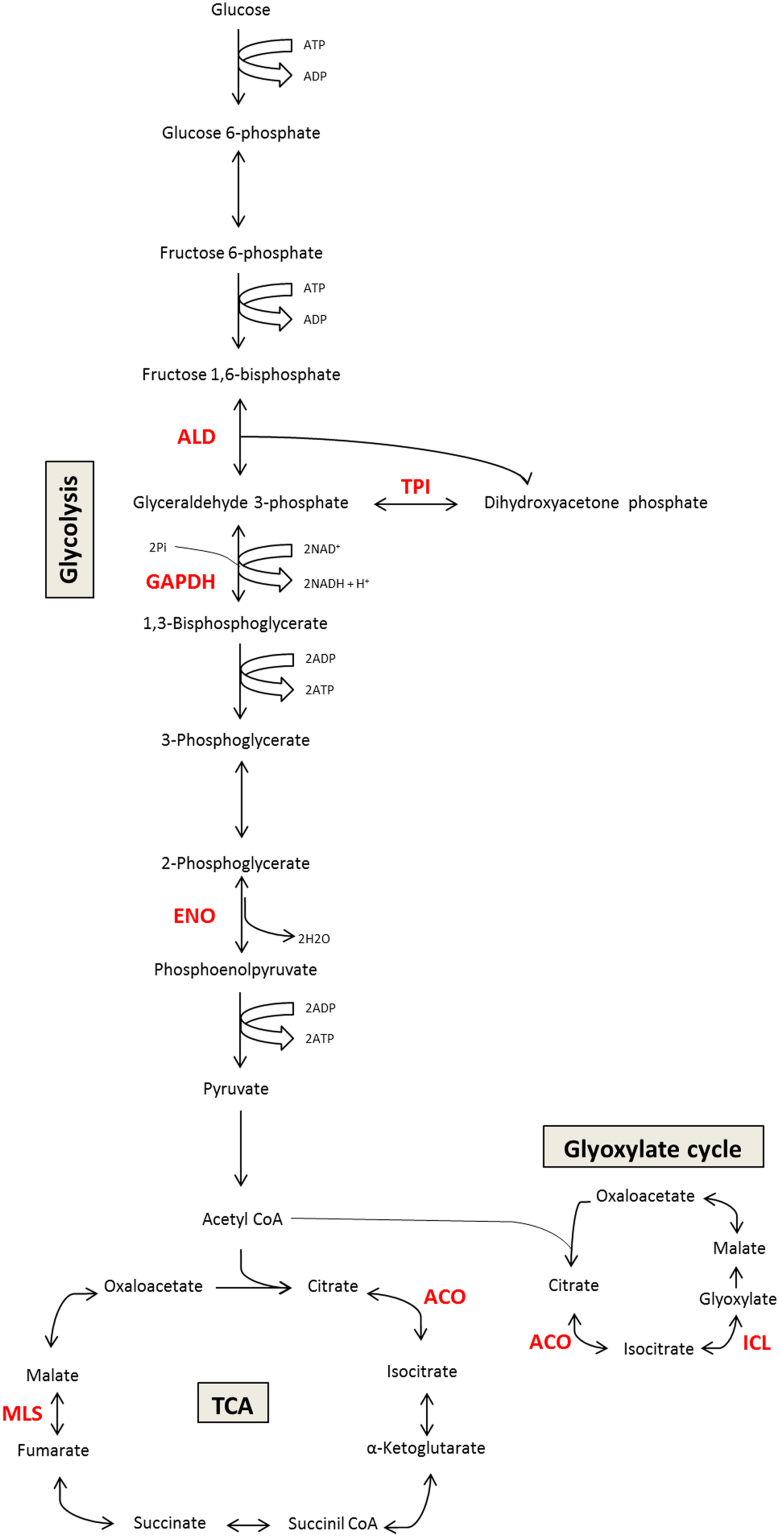
**Schematic representation of moonlighting proteins involved in *Paracoccidioides* spp. carbon metabolism (glycolysis/TCA cycle/glyoxylate cycle).** The figure summarizes the canonical function of the moonlighting proteins that are also involved in *Paracoccidioides*–host interactions. In red, we highlighted the enzymes with moonlighting functions that have been studied. ALD, aldolase; GAPDH, glyceraldehyde-3-phosphate dehydrogenase; ENO, enolase; MLS, malate synthase; TPI, triose phosphate isomerase; ACO, aconitase; ICL, isocitrate lyase.

Several studies in *Paracoccidioides* spp. show that many metabolic enzymes may play a role in virulence. The most commonly identified moonlighting functions of *Paracoccidioides* spp. include functions related to adhesion and ECM-binding activity. Enzymes in the glycolytic pathway and TCA cycle act as moonlighting proteins in *Paracoccidioides* spp, including GAPDH, ENO, and fructose-1-6-bisphosphate aldolase (FBA); each displays different affinities for binding ECM components. Additionally, malate synthase (MLS) and isocitrate lyase (ICL) from the glyoxylate pathway as well as aconitase (ACO) from the TCA cycle may have multifunctional roles, including during the interaction between the fungus and host (da Silva Neto et al., 2009; Brito et al., 2011; Cruz et al., 2011). **Figure 1** shows examples of moonlighting proteins described in *Paracoccidioides* spp.

Moonlighting functions have been increasingly recognized in glycolytic pathway and TCA cycle enzymes. In particular, despite lacking identifiable secretion signals, glycolytic enzymes have been observed on the *Paracoccidioides* surface, where they exhibit various functions that are unrelated to glycolysis, including a direct interaction with the host’s soluble proteins and surface ligands. Certain *Paracoccidioides* spp. proteins translocate to the exterior through unconventional protein secretion mechanisms, such as an affinity between certain proteins that act as transport vesicle coat components, which eventually lead to adherence or internalization and delivery to a distinct endosomal compartment in secretory vesicles (Nombela et al., 2006). This process involves vesicles derived from inward membrane invagination (endosomes), which results in protein trafficking to the plasma membrane and/or extracellular space, controlling localization and/or activity (Keller et al., 2006; Nosanchuk et al., 2008; Oliveira et al., 2010; Rodrigues et al., 2014). Straus et al. (1996) first demonstrated protein transport in vesicles in *P. brasiliensis*. Longo et al. (2014) identified many *P. brasiliensis* surface proteins in extracellular vesicles, which suggests participation of these structures in the fungal secretome and cell wall metabolism. Extracellular vesicles produced by fungi mostly contain proteins related to diverse metabolic processes. In *Paracoccidioides* spp., GAPDH, ENO, TPI and FBA were identified in the vesicle proteome and were microscopically localized to the cell wall as well as implicated in adhesion to ECM components (Vallejo et al., 2012). These results explain the reports for numerous cytoplasmic proteins, wherein the proteins perform other functions outside the plasma membrane, both in the cell wall and extracellular environment.

Another aspect for consideration is the immunological role of fungal surface proteins; these proteins may interact with the host in numerous ways and modulate the immune response (Travassos and Taborda, 2012). For example, the recognition of cell wall-associated proteins by pre-activated T cells and/or antibodies may interfere with infection (Gow and Hube, 2012). In addition, secreted proteins have important functions, such as nutrient supply, cell-to-cell communication, environmental detoxification, killing potential competitors, and aiding survival in the host (Bonin-Debs et al., 2004; Nombela et al., 2006; Holbrook et al., 2011; Weber et al., 2012). One of the main characteristics of pathogenesis is inducing damage to the host, which can occur directly due to the fungus and its virulence factors when it invades deep into or through the host tissues. Damage may also result from over-activation of the immune system through, for example, massive neutrophil infiltration or an inappropriate and unbalanced systemic response, which produces life-threatening sepsis. Thus, immune recognition may not only be beneficial and crucial for fighting invading fungi but may also be an integral part of the disease process (Gow and Hube, 2012).

## FRUCTOSE 1,6-BISPHOSPHATE ALDOLASE

Fructose 1,6-bisphosphate aldolase catalyzes reversible cleavage of fructose 1-6, bisphosphate into two triose phosphates, dihydroxyacetone phosphate and glyceraldehyde 3-phosphate. The reaction is common to glycolysis and gluconeogenesis (Marsh and Lebherz, 1992). *Paracoccidioides* spp. contains two genes that encode different Class II FBAs. FBA gene duplication in *Paracoccidioides* spp. was supported by phylogenetic analysis and established a two-member family with potentially differing functions. In addition, expression analysis support differential expression of Pbfba1 and Pbfba2, which indicates distinct functions for the two proteins (Carneiro et al., 2005). The presence of a paralogous gene supports acquisition of a new function, even if the new function is related to the original function (Tatusov et al., 1997). In *Paracoccidioides* spp., the Pbfba2 transcript was only detected in mycelial form, whereas the Pbfba1 transcript was detected in yeast cells (Chaves, 2013), further suggesting distinct functions.

Interestingly, FBA was detected in the *P. lutzii* secretome and cell wall during macrophage infection (Chaves, 2013). In proteomic studies on the yeast and mycelial phases, FBA was detected in the cell wall and extracellular vesicles, exclusively in the *P. brasiliensis* yeast-phase (Longo et al., 2014). Data show that the cell surface FBA1 includes immunogenic properties because the native protein can be recognized by serum from patients infected with paracoccidioidomycosis (PCM; da Fonseca et al., 2001). In the *Paracoccidioides* genus, both FBA isoforms could bind human plasminogen and convert plasminogen into plasmin in the presence of tissue plasminogen activator (tPA), which may increase the fibrinolytic capacity of the fungus, demonstrating that FBA is involved in the adhesion and invasion processes (Chaves, 2013). FBA also seems important in host-fungus interactions (Burucoa et al., 1995; McCarthy et al., 2002; Starnes et al., 2009; Capodagli et al., 2014).

## GLYCERALDEHYDE-3-PHOSPHATE DEHYDROGENASE

Glyceraldehyde 3-phosphate dehydrogenase is a glycolytic enzyme that catalyzes glyceraldehyde 3-phosphate conversion into 1,3-bisphosphoglycerate. The most common form in all organisms is the NAD^+^-dependent enzyme, which is typically located in the cytoplasm (Tunio et al., 2010).

In addition to the intracellular location of GAPDH, it is also present in the most external layer of the cell wall in *Paracoccidioides* spp. yeast cells at higher quantities than in the cytoplasm. The presence of GAPDH in the cell wall and extracellular vesicles of *P. brasiliensis* during the yeast and mycelium phases was demonstrated using proteomic analysis (Longo et al., 2014), which suggests that it is involved in pathogenesis (Barbosa et al., 2004). The involvement of surface GAPDH in the interaction between *Paracoccidioides* spp. and laminin, fibronectin, and type I collagen has been demonstrated (Barbosa et al., 2004, 2006). GAPDH binding to laminin, fibronectin, and type I collagen may indicate that it is a virulence factor.

Purified, recombinant GAPDH protein immunological reactivity with 70 human serum samples was tested using Western blot analysis. GAPDH reacted with antibodies in the PCM patient serum, but not in the control patients’ sera; thus, GAPDH is included in the arsenal of *P. brasiliensis* immunoreactive molecules (Santos et al., 2012).

The *Paracoccidioides* spp. GAPDH likely plays a role in the initial phases of fungal infection. A reduced interaction between *Paracoccidioides* spp. and epithelial cells was demonstrated using recombinant GAPDH protein and the polyclonal anti-GAPDH, which suggests that this protein may be important during the *Paracoccidioides* spp. infection process (Barbosa et al., 2006).

Bailão et al. (2006) used representational difference analysis (RDA) to identify genes induced during the infection process in experimental animal livers under conditions that mimic hematogenous dissemination of the fungus. The researchers showed that GAPDH was overexpressed during infection, and similar results were observed when *P. brasiliensis* yeast cells were incubated with human blood, which supports the notion that this molecule may be involved in pathogenesis. GAPDH mRNA was also identified in the *P. brasiliensis* transcriptome from mouse liver, which reinforces its potential role in the infection process (Costa et al., 2007).

In a study on the *P. brasiliensis* transcription response upon internalization by macrophages, GAPDH was down-regulated, which suggests a complex carbon-depleted environment in the macrophage that yields a similar adaptive response as in intracellular fungal pathogens (Tavares et al., 2007).

All of the data suggest that GAPDH includes adhesin characteristics and plays an important role in the fungus–host interaction, which triggers host cell processes involved in pathogenesis.

## TRIOSE PHOSPHATE ISOMERASE (TPI)

Triose phosphate isomerase (TPI) is an enzyme that rapidly interconverts dihydroxyacetone phosphate and D-glyceraldehyde 3-phosphate in the glycolysis pathway (Wierenga et al., 2010). The *Paracoccidioides* spp. TPI was first identified by da Fonseca et al. (2001) through fractionating fungus extracts using two-dimensional electrophoresis and subsequent immunoblotting. Using a strategy to identify *Paracoccidioides* spp. proteins that react with PCM patient sera, TPI was characterized as an important immunogenic molecule (Pereira et al., 2004). TPI was identified in the cell wall and extracellular vesicles using liquid chromatography coupled with high-resolution mass-spectrometry (LC-MS/MS) in the yeast and mycelium forms of two different *P. brasiliensis* isolates, Pb18 and Pb03 (Longo et al., 2014).

Additionally, TPI expression is developmentally regulated in *Paracoccidioides* spp.; expression increases when the fungus adopts the pathogenic yeast-like morphology. TPI also plays a role in the fungus–host interaction because the recombinant protein interacts with pneumocytes through binding the ECM components laminin and fibronectin. Finally, *P. brasiliensis* pre-treatment with a TPI polyclonal antibody inhibits adhesion to pneumocytes (Pereira et al., 2007).

## ENOLASE

Enolase is also referred to as phosphopyruvate hydratase and is one of the most abundantly expressed cytosolic proteins in many organisms. It is a key glycolytic enzyme that catalyzes 2-phosphoglycerate dehydration to phosphoenolpyruvate (Pancholi, 2001). ENO was identified in the cell wall and extracellular vesicles in both the *P. brasiliensis* mycelium and yeast phases; it is secreted from unconventional pathways, which was predicted using the Fungal Secretome Database (Longo et al., 2014). Unconventional extracellular export pathways include plasma membrane transporter actions and the use of vesicles that originate from the plasma membrane, lysosomal secretion, or exosome release (Nickel, 2010; Rodrigues et al., 2011).

The capacity to bind to plasminogen and fibronectin as well as superficial localization have been linked to the pathogenic role of ENO in *Paracoccidioides* spp. (Donofrio et al., 2009; Nogueira et al., 2010; Marcos et al., 2012). Certain non-glycolytic ENO properties described above, particularly the properties related to surface expression and plasminogen binding, indicate that ENO may play an important role in initiating the infection process through modulating the pericellular and intravascular fibrinolytic system. Additionally, the internal _254_FYKADEKK_262_ motif may be responsible for plasminogen binding, especially through the C-terminal lysine. *P. brasiliensis* ENO also includes an RGD motif (Arg-Gly-Asp), which is a sequence motif that mediates cell attachment. Marcos et al. (2012) demonstrated that ENO attachment to pneumocytes was inhibited, which suggests that the RGD peptide competes with the ENO binding site in pneumocytes.

Nogueira et al. (2010) demonstrated that treating epithelial cells and phagocytes with recombinant *P. brasiliensis* ENO (rPbEno) increases the effectiveness of the *Paracoccidioides* spp. interaction with host components because rPbEno enhances the exposure of surface *N*-acetylglucosamine residues, which *Paracoccidioides* spp. uses as a surface site for adherence to host cells (Coltri et al., 2006; Ganiko et al., 2007; Donofrio et al., 2009; dos Reis Almeida et al., 2010).

These data indicate that the *Paracoccidioides* spp. ENO may also have different subcellular locations (i.e., the cytoplasm or cell wall). Further, ENO has other functions in addition to its metabolic role that contributes to the virulence of this fungus.

## MALATE SYNTHASE

The glyoxylate cycle is a TCA cycle anaplerotic pathway that facilitates growth on C (2) compounds through bypassing the CO (2)-generating TCA cycle steps. MLS converts glyoxylate and acetyl-CoA to malate (Dunn et al., 2009).

In *Paracoccidioides* spp., MLS participates in the glyoxylate cycle and allantoin degradation, which allows the cell to use purine as a nitrogen source (Zambuzzi-Carvalho et al., 2009); MLS is likely important for infection because its transcript is up-regulated during the mycelium to yeast transition, during the infectious phase (Bastos et al., 2007), and, in yeast cells, during phagocytosis by murine macrophages (Derengowski et al., 2008). da Silva Neto et al. (2009) showed that the *Paracoccidioides* spp. MLS is located on the cell surface and binds certain ECM components, such as type I and IV collagens, fibronectin and pneumocytes. Anti-MLS *Paracoccidioides* spp. antibodies inhibit the interaction with epithelial cells *in vitro*, which suggests that this protein contributes to adhesion between the fungus and host tissues.

## ISOCITRATE LYASE

Isocitrate lyase is a glyoxylate cycle enzyme that converts isocitrate to glyoxylate and succinate; it is important for maintaining the TCA cycle afforded by the glyoxylate cycle when pyruvate generation from glycolysis is lower and fatty acid β-oxidation provides the major carbon source (Gould et al., 2006).

Isocitrate lyase protein was observed in the *Paracoccidioides* spp. culture filtrate; it is actively secreted to the *Paracoccidioides* spp. cell surface (Cruz et al., 2011). Troian (2009) demonstrated that recombinant ICL from *P. brasiliensis* (PbICL) and its polyclonal antibody inhibited interactions between *P. brasiliensis* and epithelial cells, which suggest a role in adhesion to host tissue. Cruz et al. (2011) reported that recombinant ICL binds fibronectin and type IV collagen, which reinforces the importance of this protein during the *Paracoccidioides–*host interaction.

Isocitrate lyase transcripts from *Paracoccidioides* spp. are induced during the yeast phase, during infection in a murine model (Felipe et al., 2005; Costa et al., 2007), and during the mycelium to yeast transition (Goldman et al., 2003; Bastos et al., 2007). Additionally, the gene that encodes ICL was induced during the fungus–macrophage interaction upon carbon starvation (Lima et al., 2014).

Argentilactone, which is a natural constituent of essential oil from *Hyptis avalifolia*, and its semi-synthetic derivate inhibited ICL activity in the presence of acetate, which affects *P. lutzii* yeast growth and mycelium to yeast differentiation (Prado et al., 2014). This finding suggests a significant role for *Paracoccidioides* spp. ICL in the host–pathogen interaction because the transition to the yeast phase of the fungus is essential for establishing infection and disease (San-Blas et al., 2002; Santana et al., 2012). Considering that pathogenic microorganisms utilize different carbon sources during pathogenesis (Barelle et al., 2006) and considering that *Pb*ICL is regulated by carbon sources (Prado et al., 2014), it is notable that ICL inhibition can affect cell growth and differentiation due a change in the carbon source used by the pathogen.

These studies indicate an adhesin behavior for *Paracoccidioides* spp. ICL and that it plays an important role in adhesion and colonization of this fungus in host tissue.

## ACONITASE

Aconitase catalyzes the second step of the TCA cycle, which includes stereo-specific isomerization of citrate to isocitrate via *cis*-aconitate. In addition to its role in energy generation, the TCA cycle generates essential precursors for amino acid, fatty acid, and carbohydrate biosynthesis (da Fonseca et al., 2001).

Brito et al. (2011) used Western blot and immunocytochemistry analysis to demonstrate *P. brasiliensis* ACO (PbACO) in the extracellular fluid and that is associated with the cell wall, mitochondria, cytosol, and peroxisomes in yeast cells. Additionally, the researchers observed that PbACO was overexpressed when the cells were grown with ethanol and acetate as carbon sources and at higher iron levels, which suggests a potential role for PbACO in iron metabolism.

## FINAL STATEMENTS

Studies on bacterial and fungal moonlighting proteins are in an early stage. One startling discovery demonstrated that the majority of proteins in the bacterial glycolytic pathway have certain adhesive functions (Henderson and Martin, 2011), which was also observed in *Paracoccidioides* spp. In this review, we collect the results for moonlighting enzyme activities in the glycolytic pathway as well as in the TCA and glyoxylate cycles, which have been described as ECM ligands in *Paracoccidioides* spp. In addition to adding to the number and types of known moonlighting proteins, these new examples also add to current information on the general importance of moonlighting proteins (Chung et al., 1999; Jeffery, 1999, 2003a, 2009).

Based on all of the data, the *Paracoccidioides* spp. moonlighting proteins include different functions in addition to their conventional metabolism roles due to their surface location. Clearly, moonlighting, or the ability to perform biological functions unrelated to the canonical function assigned to the protein, is common in fungal proteins in addition to bacteria (Kainulainen and Korhonen, 2014). A general conclusion is that moonlighting proteins seem to communicate with the environment and in response to environmental changes or stress. The moonlighting proteins present on the cell surface of *Paracoccidioides* species and released through vesicles are thought to function in host interactions. The specific roles of most cell surface proteins remain unclear, but a few such proteins are involved in cell wall biosynthesis/remodeling, adaptation to different environmental conditions, and PCM pathogenesis. Thus, moonlighting proteins may be potential targets for designing drugs against systemic mycosis.

## Conflict of Interest Statement

The authors declare that the research was conducted in the absence of any commercial or financial relationships that could be construed as a potential conflict of interest.
